# A tRNA- and Anticodon-Centric View of the Evolution of Aminoacyl-tRNA Synthetases, tRNAomes, and the Genetic Code

**DOI:** 10.3390/life9020037

**Published:** 2019-05-04

**Authors:** Yunsoo Kim, Kristopher Opron, Zachary F. Burton

**Affiliations:** 1 University of Michigan, Ann Arbor, MI 48109, USA; yunsoo@umich.edu; 2 Bioinformatics Core, University of Michigan, Ann Arbor, MI 48109-0674, USA; kopron@gmail.com; 3 Department of Biochemistry and Molecular Biology, 603 Wilson Rd., Michigan State University, East Lansing, MI 48824-1319, USA

**Keywords:** anticodon, aminoacyl-tRNA synthetase, coevolution theory, “frozen accident”, genetic code, glycine, Phyre2, polyglycine, tRNAome, tRNA evolution

## Abstract

Pathways of standard genetic code evolution remain conserved and apparent, particularly upon analysis of aminoacyl-tRNA synthetase (aaRS) lineages. Despite having incompatible active site folds, class I and class II aaRS are homologs by sequence. Specifically, structural class IA aaRS enzymes derive from class IIA aaRS enzymes by in-frame extension of the protein N-terminus and by an alternate fold nucleated by the N-terminal extension. The divergence of aaRS enzymes in the class I and class II clades was analyzed using the Phyre2 protein fold recognition server. The class I aaRS radiated from the class IA enzymes, and the class II aaRS radiated from the class IIA enzymes. The radiations of aaRS enzymes bolster the coevolution theory for evolution of the amino acids, tRNAomes, the genetic code, and aaRS enzymes and support a tRNA anticodon-centric perspective. We posit that second- and third-position tRNA anticodon sequence preference (C>(U~G)>A) powerfully selected the sectoring pathway for the code. GlyRS-IIA appears to have been the primordial aaRS from which all aaRS enzymes evolved, and glycine appears to have been the primordial amino acid around which the genetic code evolved.

## 1. Introduction

Surprisingly, a sequence record has been maintained of some of the first and most central events in evolution of life on Earth. The record can be read in the sequences of tRNAs and aminoacyl-tRNA synthetases (aaRS; i.e., GlyRS-IIA (structural subclass IIA)) [1,2,3,4,5]. tRNAs are charged with amino acids by aaRS enzymes, so aaRS were some of the first enzymes to evolve [6]. Because the record was written in languages (RNA and protein sequences) that can be read, a historical record is apparent of events on Earth from ~4 billion years ago. Remarkably, the sequence of the primordial tRNA (tRNA^Pri^) is known almost to the last nucleotide, and the primordial sequence remains detectable in the tRNAs of ancient archaea [3,4,7]. The primordial tRNA core sequence (i.e., 1–75; lacking 3′-ACCA) was generated entirely from repeating sequences and inverted repeats (stem-loop-stems). In the ancient world before cells, in order to replicate RNAs, inverted repeats were ligated to RNAs as snapback primers for complementary strand replication [8]. The primordial tRNA radiated to form tRNAomes, which are the entire sets of tRNAs for an organism [1].

Most tRNAs are of type I, but some are type II (i.e., tRNA^Leu^ and tRNA^Ser^), with expanded variable (V) loops [9,10]. Both types of tRNA are described by the same, simple evolution model. The radiations of aaRS enzymes closely track the evolution of the genetic code and describe the sectoring and structuring of the code, which evolved according to rules of sequence preference for the tRNA anticodon. A remarkably detailed working model for evolution of the genetic code, therefore, can be inferred. With a small number of simplifying assumptions, the evolution of mRNA can be described based on tRNA and genetic code evolution. Ribosomes and rRNA coevolved with translation systems, particularly around tRNA [5]. Taking a reductionist evolutionary view based on existing sequences, translation systems of apparently overwhelming complexity appear simple.

Before evolution of proteins, tRNAs and minihelices were probably charged with ribozyme aaRS enzymes [11,12,13]. Because, when the genetic code was first established, protein aaRS enzymes replaced ribozyme aaRS, protein aaRS are among the oldest encoded proteins [6,14]. Analyzing sequences and structures of aaRS enzymes, particularly in ancient archaea, the pathways of aaRS enzyme radiation were determined with little or no ambiguity. Notably, two classes of aaRS enzymes were identified with distinct and incompatible active site folds. Class II aaRS appear older than class I aaRS enzymes. Class II enzymes have an active site of antiparallel β-sheets with conserved active site motifs 1, 2, 2b, and 3. Class I enzymes have an active site of parallel β-sheets described as a Rossmann fold or a Rossmannoid fold. Because no evolutionary relationship exists between class I aaRS and (α−β)_8_ Rossmann fold proteins (i.e., dehydrogenases), however, the Rossmannoid designation is better. Multiple structural classes IIA–D and IA–C have been characterized [6,14,15]. GlyRS-IIA appears to be closest to the primordial aaRS. Interestingly, despite their alternate folds, ValRS-IA and IleRS-IA enzymes have been shown to be sequence homologs of GlyRS-IIA [2]. The class I aaRS fold is derived by extending the N-terminus of a class IIA enzyme via an in-frame upstream transcription and translation start and by refolding of the aaRS structure nucleated by the N-terminal extension, which forms part of the class I aaRS active site. Initially, Zn fingers were of particular importance in directing the alternate class IIA and class IA folds [2,5]. All aaRS enzymes, therefore, radiated from GlyRS-IIA.

For specificity, aaRS enzymes recognize the tRNA, the amino acid, and ATP [6]. On tRNA, aaRS recognize: (1) the acceptor stem; (2) the anticodon loop; (3) the “discriminator” base (5′-NCCA-3′; N is the discriminator); (4) sometimes specific tRNA modifications; (5) other determinants in cognate tRNA; and (6) antideterminants in non-cognate tRNAs [6]. Class I and class II aaRS bind to opposite faces of the acceptor stem and tRNA. In the aaRS synthetic active site, the amino acid is covalently adenylated, releasing pyrophosphate, and then the activated amino acid (aa-AMP) is transferred to the tRNA 3′-CCA adenine ribose ring, releasing AMP. Some aaRS have separate editing active sites that hydrolyze inaccurately attached amino acids. In archaea, editing active sites on aaRS are limited to hydrophobic and neutral amino acids, indicating that amino acids with more character can more easily be discriminated in the aaRS active site [2,5]. Ribozyme aaRS generated in vitro utilize aa-AMP as a substrate [11,12,16]. Other ribozymes generated in vitro adenylate amino acids [17,18].

## 2. Materials and Methods

### 2.1. Homology of GlyRS-IIA and IleRS-IA and GlyRS-IIA and ValRS-IA

To search for the closest matches comparing GlyRS-IIA and IleRS-IA and GlyRS-IIA and ValRS-IA, we followed the National Center for Biotechnology Informatics (NCBI) protocol: https://www.ncbi.nlm.nih.gov/tools/gbench/tutorial18/. For multiple sequence alignments (not shown) the following sequences were compared: WP_011012235.1; WP_011011404.1; WP_011012774; WP_011839420; WP_009988726; WP_011838370; WP_011011404; WP_011839031; Q8ZWU4; WP_081225707; WP_096204125.1; WP_011500185.1; WP_071907205.1; WP_095652122.1; 4KR2_A; 4KQE_A; 1GAX_A. Using these sequences, our alignments can be reproduced using NCBI Blast tools. The resulting multiple sequence alignment is the same as previously reported and supports the schematic model previously reported [2].

### 2.2. Determining Kinship among aaRS Enzymes

Phyre2 was used to search the Protein Data Bank for the nearest relatives of aaRS enzymes [19]. For most searches, the query sequence was from *Pyrococcus furiosis*, which is an ancient archaea that is closely related to the last universal common (cellular) ancestor (LUCA) for translation functions [1,2,4,5]. *Pyrococcus* lacks GlnRS-IB (*Saccharomyces cerevisiae*), pSerRS-IIC (*Archaeoglobus fulgidus*), LysRS-IIB (*Thermus thermophilus*), GlyRS-IID (*Escherichia coli*), and pyrroLysRS-IIC (*Methanosarcina mazei*), so these were obtained from other species as indicated. Alignments with low scores were checked to ensure that homologous domains and residues were properly aligned. All alignments appeared to be accurate, even alignments with much lower scores than those reported here. Many alignments reliably obtained using Phyre2 could not be obtained using sequence searching tools such as NCBI Blast. Phyre2 utilizes sequence- and structure-matching tools, so Phyre2 is only useful to identify the relatedness of class I to other class I aaRS enzymes or else class II to other class II aaRS enzymes. Because of the different class I and class II folds, Phyre2 cannot be used to demonstrate homology of class I aaRS enzymes to class II aaRS enzymes, so this was done using sequence-searching methods. The Phyre2 server is a very easy and inexpensive bioinformatics approach for anyone to use to identify the homology of proteins with structural similarity but low sequence similarity.

### 2.3. Molecular Graphics

Molecular graphics were developed using University of California, San Francisco Chimera [20].

## 3. The Hypothesis

We offer a set of nested hypotheses that emanate from a central hypothesis. The core idea is that, because tRNA is the central molecule in molecular coding, tRNA is the most essential molecule in evolution of life on Earth. If this central idea is accepted and pursued, many related ideas arise, making evolution of life on Earth into a surprisingly straightforward story and a story that is preserved in code. For clarity, the overall set of hypotheses is described here and then reprised at the end of the review with an explanatory figure. We posit that the genetic code, translation systems, and mRNA evolved around tRNA. The meaning of this hypothesis is that tRNA is the central innovation and driver of the evolution of life on Earth. Viewed as intellectual property, tRNA is the major advance that allows genetic coding and, therefore, makes possible proteins of a defined sequence, thus enabling the evolution of biological complexity. tRNA has unique properties that make it suitable as a translation adapter. Based on order detected in tRNA sequences, we posit that tRNA is the product of RNA- and ribozyme-driven abiogenesis, the process by which chemistry drives early evolution of pre-living systems before the DNA/RNA-protein world and before the advent of cellular organisms. We show that tRNA evolved from repeat sequences and inverted repeats (stem-loop-stems), providing insight into a strange polymer world and minihelix world that preceded tRNA. A handful of ribozymes that have been generated in vitro appear sufficient to generate tRNA, indicating that abiogenesis of tRNA is possible. Abiogenesis of tRNA leads to the DNA/RNA-protein world, which evolved to a complete genetic code. Ultimately, the DNA/RNA-protein world evolved to the last universal common (cellular) ancestor (LUCA), intact DNA genomes, and cellular life. Based on tRNA sequences, we posit that glycine was the primordial amino acid charged to minihelices and to tRNAs, initially via a ribozyme GlyRS [11,12], before evolution of a protein GlyRS enzyme that required a mature genetic code. The genetic code, therefore, initially evolved using any mRNA sequence to encode polyglycine. We posit that, in the ancient world, polyglycine was utilized to stabilize protocells [21,22,23,24], much as polyglycine currently stabilizes bacterial cell walls [25]. We posit that synthesis of polyglycine to stabilize protocells was the initial selection for the primitive genetic code. We posit that the genetic code, usually shown as a 64-letter code in mRNA, can be reduced to a 32-letter code in tRNA [1,2,4,5]. After filling the genetic code with glycine anticodons, the code evolved via invasion and competition to encode 20 amino acids and stops, as we describe below. According to such a model, incoming amino acids displaced previously encoded amino acids. Established amino acids within a primitive code competed and won against newly encoded amino acids such that established amino acids retained more favored code sectors. We provide evidence that the genetic code evolved around the tRNA anticodon. We derive rules to describe the apparent pathway for sectoring of the genetic code to its current form, based on the tRNA anticodon and its interpretation on the ribosome [5]. Amino acids, tRNA, mRNA, aaRS enzymes, the genetic code, and the ribosome are posited to be coevolved, as described by the coevolution hypothesis [26,27,28]. Analysis of aaRS and tRNAome coevolution with the genetic code shows that the code evolved rapidly and mostly irreversibly via a “frozen accident”, as hypothesized by Francis Crick [26,27,28,29]. Because the LUCA was present ≥3.85 billion years ago and the first evidence of pre-life is from ~4.1 billion years ago (or earlier), the evolution of the genetic code appears to fit into a tight window of a few hundred million years [30,31,32].

## 4. Evolution of tRNA

### 4.1. A Model for tRNA Evolution

**Figure 1:**
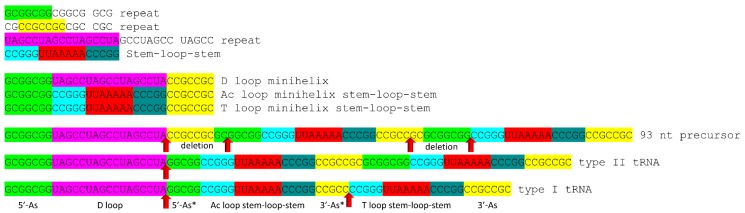
Evolution of tRNA. We posit that tRNA evolved from repeats and inverted repeats generated by RNA- and ribozyme-driven abiogenesis. A strange polymer world leads to a minihelix world to a tRNA world with type I and type II tRNAs. A 93-nucleotide tRNA precursor (3 × 31 nucleotides) is deleted internally by nine nucleotides, once to form type II tRNAs and twice to form type I tRNAs, as shown (red arrows).

**Figure 2:**
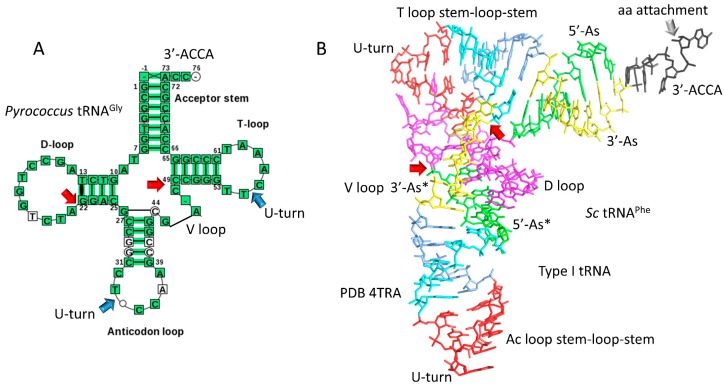
Type I tRNA. (**A**) A typical type I tRNA diagram [9] of tRNA^Gly^ from *Pyrococcus* (three species). (**B**) A type I tRNA colored for internal homologies as in Figure 1 (*Saccharomyces cerevisiae* (*Sc*) tRNA^Phe^) [33]. Ac, anticodon. Blue arrows indicate U-turns. Red arrows indicate positions of internal deletions processing the 93-nucleotide precursor (magenta→green and yellow→cyan) (Figure 1). The grey arrow indicates the site of amino acid attachment.

**Figure 3:**
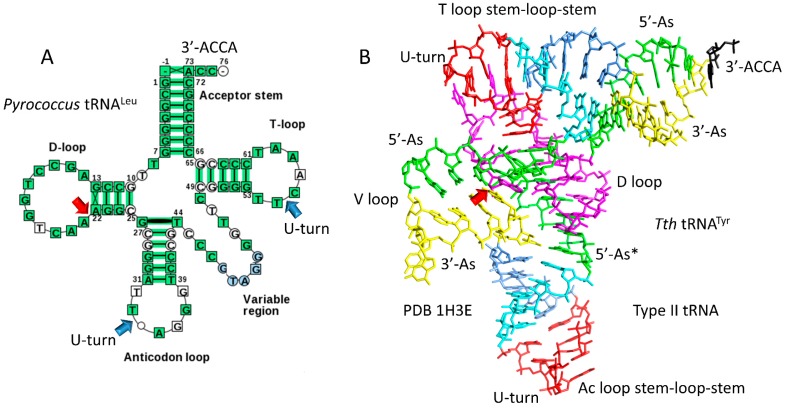
Type II tRNA. (**A**) A typical type II tRNA diagram [9] of tRNA^Leu^ from *Pyrococcus* (three species). (**B**) A type II tRNA colored for internal homologies as in Figure 1 (*Thermus thermophilus (Tth)* tRNA^Tyr^) [34]. Ac, anticodon. Blue arrows indicate U-turns. Red arrows indicate positions of internal deletions processing the 93 -nucleotide precursor (magenta→green) (Figure 1).

**Figure 4:**
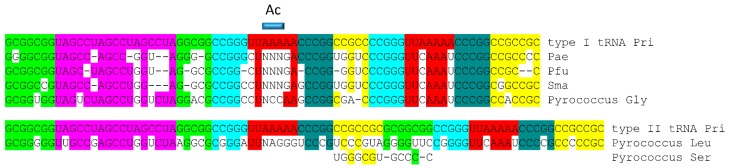
A comparison of typical tRNA sequences and tRNA^Pri^ (type I and type II). “–“ indicates that no specific sequence was selected for the typical tRNA although a base is (or may be) present. “N” indicates A, G, C, or U (A is not utilized in the anticodon wobble position in archaea [2,35]). Pae, *Pyrobaculum aerophilum*; Pfu, *Pyrococcus furiosis*; Sma, *Staphylothermus marinus*, Ac, anticodon. For tRNA^Ser^, only the first seven and last seven nucleotides of the tRNA^Ser^ variable loop are shown [4].

Sequences of tRNAs in archaea strongly support a model for tRNA evolution (Figure 1). Using statistical tests, the same model can be justified for bacterial tRNAs, which are more highly derived in evolution [3,4]. The same model describes evolution of primordial type I and type II tRNA (tRNA^Pri^) [3,4,7]. Type I tRNA are described in Figure 2, and type II tRNA are described in Figure 3. Figure 4 shows the similarities in sequence of tRNA^Pri^, typical tRNA^Gly^, and typical tRNAs from ancient archaea. tRNA evolved from GCG, CGC, and UAGCC repeats, which, remarkably, remain conserved in archaeal tRNA sequences. The primordial 5′-acceptor stem sequence is GCGGCGG, a truncated GCG repeat. The 3′-acceptor stem sequence was initially CCGCCGC, a complementary, truncated CGC repeat. The D loop sequence was initially UAGCCUAGCCUAGCCUA, a truncated UAGCC repeat. The anticodon loop and the T loop were initially identical, with a primordial stem-loop-stem sequence of ~CCGGGUUAAAAACCCGG. The only slight sequence ambiguity is in the seven-nucleotide loop, not in the 5-nt complementary stems, which are conserved and apparent in archaeal tRNAs (Figure 4). The ~UUAAAAA loop forms a U-turn after the second U. The U-turn forms a compact seven-nucleotide loop that aligns ~AAAAA (the first AAA comprise the three-nucleotide anticodon in the Ac loop) in a helical conformation in line with the CCCGG 3′-stem. Without the relatively stiff 7-mer loop, tRNA would not constitute an adequate adapter for translation. Between the D loop and the anticodon stem-loop-stem is a five-nucleotide fragment of a 5′-acceptor stem with the primordial sequence GGCGG, which was formed by internal RNA processing. In type I tRNAs, between the anticodon stem-loop-stem and the T loop stem-loop-stem is a fragment of a 3′-acceptor stem with the primordial sequence CCGCC, which was also formed by internal RNA processing. CCGCC becomes the five-nucleotide V loop in type I tRNAs (Figure 1 and Figure 2). In type II tRNAs (Figure 1 and Figure 3), the expanded V loop results from a ligated 3′-acceptor stem and a 5′-acceptor stem with the primordial and unprocessed sequence CCGCCGCGCGGCGG. Because the CCGCC sequence in type I tRNAs is a fragment of CCGCCGCGCGGCGG, after nine-nucleotide deletion, the type I CCGCC sequence is the product of an internal deletion. Similarly, the 5′-acceptor stem remnant GGCGG that became the last five nucleotides of the D loop, before the anticodon 5′ stem, is a product of internal nine-nucleotide deletion.

In summary, type I and type II tRNAs evolved as follows (Figure 1). Here, 3–31 nucleotide minihelices were ligated to form a 93-nucleotide tRNA precursor. An internal deletion of nine nucleotides within the sequence CCGCCGCGCGGCGG left GGCGG, the last five nucleotides of the D loop region before the 5′-anticodon stem. In type I tRNAs, an internal deletion of nine nucleotides within the sequence CCGCCGCGCGGCGG left the sequence CCGCC, which became the V loop. In type II tRNAs, the sequence CCGCCGCGCGGCGG was maintained with no processing, forming the first iteration of the expanded V loop. Type II tRNAs, therefore, formed from a predicted intermediate in the formation of type I tRNAs, and, furthermore, the same model describes evolution of type I and type II tRNAs. Generation of tRNAomes, which are the complete sets of tRNAs for an organism, resulted in many sequence changes in tRNAs, in order to specify attachments of different amino acids. Every aspect of this detailed evolution model is supported by statistical tests [3,4]. Conserved deviations in typical tRNAs from tRNA^Pri^ in Figure 4 can largely be explained by selective pressures of tRNA folding [3,4,5].

Alternate models have been advanced to describe tRNA evolution based on ligation of two minihelices instead of three [36,37,38,39,40,41]. These models are critically flawed. First, in a two minihelix model the anticodon loop and the T loop cannot be homologs, as we show they certainly are (i.e., Figure 2A and Figure 4) [1,3,4]. Second, two minihelix models may predict a relationship (homology or complementarity) of the D loop region and the T loop region, which cannot be demonstrated.

Interestingly, A is very rare in the archaeal and bacterial tRNA anticodon wobble position [2,35]. In bacteria, A is essentially only encoded when A is converted to inosine by deamination (i.e., bacterial tRNA^Arg^(ACG→ICG) (I for inosine)). So, A is strongly disfavored in the anticodon wobble position in prokaryotes. We posit that G is favored over A, because a G~U wobble pair is more stable than an A~C wobble pair, and, at the wobble position, only purine versus pyrimidine resolution is generally achieved in prokaryotes [1,2,4,5]. Because A is disfavored in the wobble position, the size of the genetic code is smaller in tRNA than in mRNA. Because U and C are difficult to distinguish in the tRNA wobble position, this shrinks the size of the genetic code further in prokaryotes, near the base of code evolution. We do not know when in evolution the strong selection against wobble A appeared. In this review, we posit that A is also disfavored in the third anticodon position (see below).

### 4.2. Evolution from Order to Chaos

Because tRNA^Pri^ evolved from repeats and inverted repeats (Figure 1 and Figure 4), mechanisms existed to generate ordered sequences before evolution of translation systems. Repeats can be synthesized via abortive initiation followed by ligation or via replication slippage without dissociation. Telomerase extends and maintains long repeat sequences utilizing an enzyme-associated RNA template (TERT for telomerase reverse transcriptase), so a telomerase-like ribozyme carrying a mobile RNA template, generating repeating sequences, may be possible. A reverse transcriptase and RNA polymerase ribozyme has been generated in vitro [42]. Order in tRNA sequences shows that not all early polymerization reactions resulted in random polymer assembly. Some early polymers were ordered as repeats and inverted repeats before abiogenic processes were replaced by cellular life. Because living systems evolved around tRNAs, tRNA^Pri^, which had a highly ordered sequence, was the crowning achievement of abiogenesis.

### 4.3. Evolution around the tRNA Anticodon

**Figure 5:**
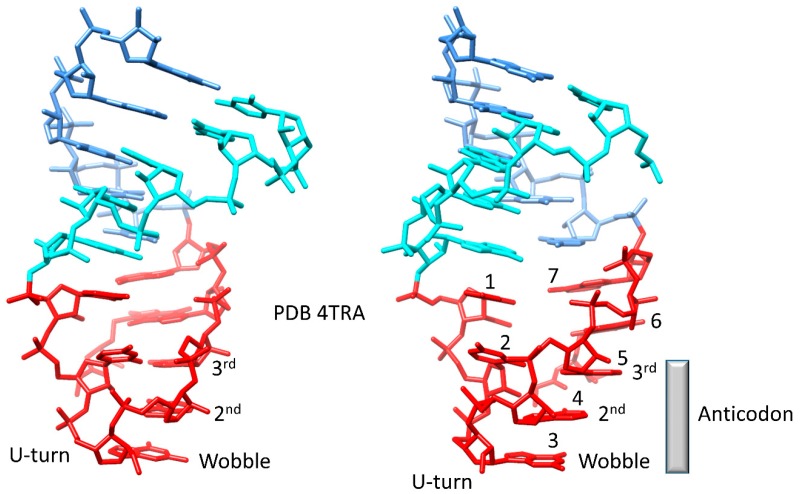
The tRNA anticodon stem-loop-stem from PDB (Protein Data Bank) 4TRA [33]. The 5′ stem is cyan, the loop is red, and the 3′ stem is cornflower blue. Loop positions 1–7 and anticodon positions are labeled. Two views are shown.

The genetic code evolved primarily around tRNA rather than around mRNA, ribosomes, or aaRS enzymes [1,2,4,5]. Because of the primacy of tRNA, the tRNA anticodon, and the manner by which the anticodon is read on the ribosome are central guides to paths of code evolution. In Figure 5, the anticodon stem-loop-stem is shown. The conformation of the anticodon loop in the figure is very similar to the conformation on a translating ribosome. The tRNA anticodon loop is of seven nucleotides, although there are typically weak (non-Watson–Crick) interactions between loop bases 1←→7 and 2←→6. The loop cannot be six or eight nucleotides, because a U-turn could not then form between loop positions 2 and 3. The U-turn is important because this aligns loop positions 3–7 with the 3′-anticodon stem, as if in a helical conformation (Figure 5) [43]. The compact seven-nucleotide loop with a U-turn separating loop bases 2 and 3, therefore, is the only RNA loop configuration for an adequate and relatively stiff adapter, presenting a 3-nucleotide anticodon. Because of the manner by which the ribosome reads the tRNA anticodon, the second anticodon position is most important for translational accuracy followed by the third anticodon position. During translation, Watson–Crick geometry is forced on the second and third anticodon positions, allowing these positions to be read with single base resolution and discrimination (i.e., A, G, C, and U are accurately discriminated at anticodon positions 2 and 3) [44,45,46,47]. The wobble position of the tRNA anticodon, by contrast, cannot easily be read with single base accuracy. Rather, at the base of code evolution, only purine/pyrimidine discrimination (i.e., A/G versus C/U) is practical at the wobble position. The genetic code, therefore, read in tRNA, is a 32-letter code versus a 64-letter code in mRNA [2,4,5]. Ultimately, restrictions in accurately reading tRNA describe why a genetic code that might be considered to encode as many as 63 amino acids in mRNA encodes only 20 amino acids in tRNA.

### 4.4. Addition of 3′-ACCA

A full explanation of tRNA structure and function requires 3′-addition of 5′-ACCA-3′, the sequence to which the amino acid is attached to the ribose ring of 3′-A. The 5′-A of ACCA is referred to as the “discriminator” base, which becomes very important for accurate attachment of amino acids by aaRS enzymes [6,14,15]. In archaea, however, many or most tRNAs utilize discriminator A, indicating that A was the first discriminator base at the base of code evolution [9,10]. In some organisms, ACCA is encoded in tRNA genes, and in others, the discriminator is encoded in the tRNA gene but CCA is added enzymatically. We posit that 3′-ACCA was initially added to tRNAs and also to minihelices perhaps by ligation. Subsequently, the discriminator base and sometimes CCA became incorporated as part of tRNA genes. The ancient world included reverse transcriptase activities, so sequences added to tRNAs could become sequences in genes.

## 5. Glycine as the Primordial Amino Acid

From sequence, tRNA^Pri^ appears to be a primordial tRNA^Gly^ (Figure 1, Figure 2A, and Figure 4). Furthermore, a typical tRNA from ancient archaea resembles tRNA^Pri^ and tRNA^Gly^ (Figure 4). Based on these observations, tRNA^Pri^ appears to have been an ancestral tRNA^Gly^, and glycine appears to be the primordial amino acid around which tRNAomes and the genetic code evolved [1,2,4,5,48,49,50]. Based on these ideas, we assumed that tRNA^Gly^ filled the entire genetic code table at an early stage of evolution. This assumption was simplifying, because it provided a Darwinian selection for mRNA and tRNA to coevolve to generate the genetic code. If this assumption is not accepted, for instance, if the genetic code is filled from a corner, a sector at a time, coevolving mRNA and tRNA becomes a much more challenging problem.

The peptidoglycan layer of bacterial cell walls is formed of polyglycine (i.e., Gly5) and a glycan chain (i.e., a GlcNAc-[1→4]-MurNAc polymer) modified on MurNac 3′-O with a chain of amino acids (i.e., L-Ala-D-iso-Gln-L-Lys-D-Ala-D-Ala) to which the Gly5 chain is N-terminal cross-linked to D-Ala and C-terminal bridge-linked to a second identical peptide chain at L-Lys [25]. The GlcNAc-MurNAc polymer anchors covalently to membrane lipids. Assuming that a similar structure stabilized protocells ~4 billion years ago, polyglycine would have been of evolutionary value before evolution of the genetic code and before cellular organisms with intact genomes. tRNA^Pri^ and the genetic code, therefore, are posited to have evolved initially to synthesize polyglycine as an improved mechanism to stabilize protocells. From such a beginning, clear mechanisms can be posited to evolve the genetic code encoding proteins.

## 6. Coevolution of aaRS Enzymes and the Genetic Code

### 6.1. Evolution of Class II aaRS

**Figure 6:**
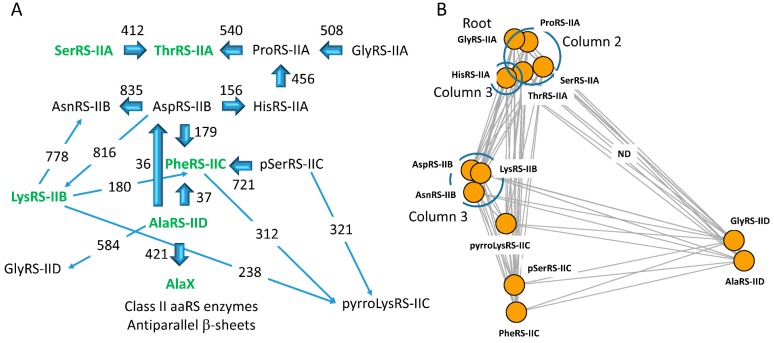
Homology and radiation of class II aminoacyl-tRNA synthetase (aaRS) enzymes. Two representations of the network are shown. (**A**) A hand-drawn version. Enzymes that have editing active sites are in green and bold type. The direction of the arrows is from the query→target aaRS, although comparisons were generally done in both directions. The numbers reported are the Phyre2 homology scoring function [19]. The larger the number, the greater the homology. In most cases, the directionality of the arrows and the score indicates the highest score obtained using Phyre2 homology searches. Of the editing aaRS fragments, only AlaX is considered because AlaX is the only editing enzyme (missing a synthetic active site) detected in *Pyrococcus furiosis*. AlaX is only homologous to the editing domain of AlaRS-IID and no other aaRS. (**B**) Igraph representation in which distances between nodes represent the evolutionary distance (Kamada and Kawai settings) [51]. The program has drawn lines for relatedness in which homology was not detected (ND) (Table 1). Circles indicate related aaRS enzymes within genetic code columns. pSer, *o*-phosphoseryl; pyrroLys, pyrrolysyl.

Figure 6 shows a proposed lineage of class II aaRS enzymes based on the Phyre2 protein structure recognition server searches. Mostly, *Pyrococcus furiosis* aaRS enzymes were used as the search query. *P. furiosis* was selected because, for translation systems, *P. furiosis* is an ancient archaea that is similar to LUCA [1,2,5]. The apparent pathway is similar to that we previously reported [2,5] but with key improvements and more reliable connections. Notably, there is little or no ambiguity in the networks reported here.

Two representations of the network are shown. Figure 6A shows a hand-drawn network with closest genetic distances indicated as Phyre2 homology scores. Figure 6B shows an Igraph representation in which distances between nodes represent evolutionary distances. As we anticipated, the lineage appears to root to GlyRS-IIA. Connections in the lineage are quantified using the Phyre2 scoring metric in which, the larger the score, the closer the homology [19]. The weakest node connections in the lineage are from HisRS-IIA to AspRS-IIB (score 156, e-value 1.3 × 10^−22^), from AspRS-IIB to PheRS-IIC (score 179; e-value 1.6 × 10^−26^), and from PheRS-IIC to AlaRS-IID (score 37; e-value 0.017) or AspRS-IIB to AlaRS-IID (score 36; e-value 0.022). PheRS-IIC is unlikely to be derived from LysRS-IIB because LysRS-IIB is a bacterial innovation that is missing from archaea, in which LysRS-IE is present [6,15]. We posit that archaeal LysRS-IE must be the older enzyme variant. All other nearest connections in the class II aaRS lineage are robust and unambiguous. With the exceptions of PheRS-IIC to AlaRS-IID and AspRS-IIB to AlaRS-IID, close connections are long alignments with conserved active site motifs 1, 2, 2b, and 3 aligned. The weak connections to AlaRS-IID, therefore, are the only slight uncertainties in the map. All of the class II aaRS homology scores obtained are shown in Table 1. Although all class II aaRS are homologs, not all are connected by detectable homology using the Phyre2 server. AlaRS-IID and GlyRS-IID are only weakly homologous to other class II aaRS enzymes. GlyRS-IID is a bacterial innovation found in some bacterial species. Only GlyRS-IIA is found in archaea.

Homologous aaRS enzymes tend to arrange in genetic code columns indicating co-evolution of aaRS enzymes and the genetic code [2,4,5,27,28]. In Figure 6B, circles are drawn around related aaRS enzymes that group within genetic code columns. In archaea, AspRS-IIB and AsnRS-IIB are closely related, and HisRS-IIA is related. In bacteria, AspRS-IIB, AsnRS-IIB and LysRS-IIB are closely related, and HisRS-IIA is related. Archaea utilize LysRS-IE. In bacteria, LysRS-IIB may have evolved from AspRS-IIB.

A homology was detected comparing some AlaRS-IID (*Pyrococcus furiosis*) and ThrRS-IIA (*Staphylococcus aureus* (strain MW2)) structures (Phyre2 score 204; e-value 2.4 × 10^−26^). The structure and sequence similarity, however, was discounted for the current analysis. It was clear that the shared domain did not include the active site. The shared domain was part of a later genetic swap that did not relate to the early radiation of aaRS enzymes. There is no clear homology comparing *P. furiosis* AlaRS-IID and *P. furiosis* ThrRS-IIA, indicating that, near the base of aaRS evolution, AlaRS-IID and ThrRS-IIA are not closely related and do not both include the detected homologous domain that was shared later in evolution.

### 6.2. Evolution of Class I aaRS

**Figure 7:**
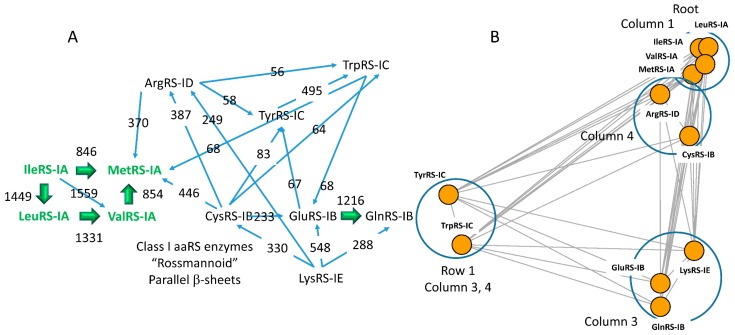
Homology and radiation of class I aaRS enzymes. Two representations of the network are shown. (**A**) A hand-drawn representation. Enzymes that have editing active sites are in green and bold type. The direction of the arrows is from the query→target aaRS. The numbers reported are the Phyre2 homology scoring function (Table 2). The larger the number, the better the match. The directionality of the arrows generally indicates the highest score obtained in Phyre2 homology searches, which were done in both directions. (**B**) Igraph representation. Distances represent evolutionary distances (Kamada and Kawai settings) [51]. Circles indicate related aaRS enzymes grouped in genetic code columns and rows.

The apparent lineage of class I aaRS enzymes is shown in two representations (Figure 7). Figure 7A shows a hand-drawn network. Figure 7B shows an Igraph representation in which distances between nodes represent evolutionary distances. Class IA enzymes ValRS-IA, IleRS-IA, LeuRS-IA, and MetRS-IA are very closely related, and the class I aaRS lineage appears to root to class IA aaRS enzymes, as we expected. Because class I aaRS enzymes are about twice as long as class II aaRS enzymes, alignments are longer, and the highest scores are therefore larger for class I aaRS enzyme homologies. The weakest connections in the map are to TyrRS-IC and TrpRS-IC, which are closely related to one another. We posit that TyrRS-IC may be most closely related to an ancestor of CysRS-IB (score 83; e-value 1.7 × 10^−9^), although other homologies with comparable scores are evident. Inspection of the TyrRS-IC to CysRS-IB alignment shows that it is long and robust, as indicated by the e-value. In most alignments reported, conserved active site motifs HIGH and KMSKS align, indicating that the alignments are reliable and accurate [15]. Phyre2 homology scores are shown in Table 2. In the class I aaRS evolutionary map, all class I aaRS are connected to one another by detectable homology. Many of these homologies would not be obtained using sequence-based alignment methods rather than Phyre2, which utilizes both sequence and structure. In Figure 7B, circles are drawn around related aaRS enzymes that are located in genetic code columns and in rows and neighboring columns. Closely related ValRS-IA, IleRS-IA, MetRS-IA, and LeuRS-IA are found clustered in column 1 of the genetic code table. Closely related GluRS-IB, GlnRS-IB, and LysRS-IE are found in column 3 of the genetic code table in rows 4B, 3B, and 2B. Closely related CysRS-IB and ArgRS-ID are found in column 4 of the genetic code table. Closely related TyrRS-IC and TrpRS-IC are found in row 1 of the genetic code table in neighboring columns 3 and 4.

### 6.3. Homology of Class I and Class II aaRS

**Figure 8:**
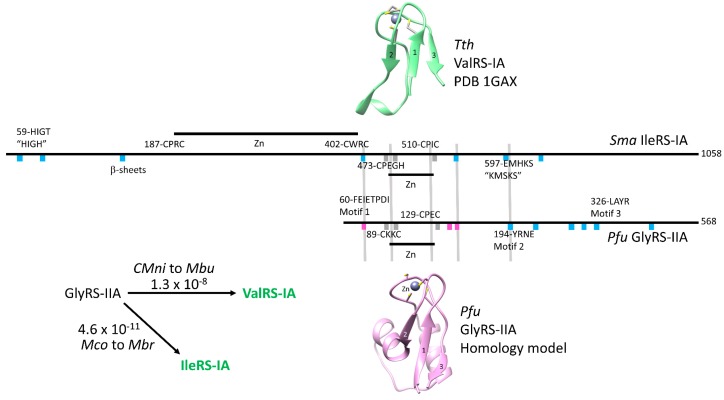
A schematic sequence alignment of a class IIA aaRS and a class IA aaRS is shown [2,5]. The shared Zn fingers are shown as structures. Cyan blocks indicate active site β-sheets. Grey blocks indicate shared β-sheets that organize Zn fingers. Magenta blocks indicate β-sheets surrounding the Zn finger in *Pyrococcus furiosis* (*Pfu*) GlyRS-IIA that are organized by the Zn finger. *Tth* indicates *Thermus thermophilus*. NCBI tools were used to find close matches comparing GlyRS-IIA and IleRS-IA and GlyRS-IIA and ValRS-IA, in order to demonstrate homology of class I and class II aaRS enzymes. e-values are shown. The species compared are indicated: *Candidatus Methanoperedens nitroreducens* (*CMni*), *Methanococcoides burtonii* (*Mbu*), *Methanobacterium congolense* (*Mco*), and *Methanobacterium bryantii* (*Mbr*). Green type indicates ValRS-IA and IleRS-IA have editing active sites.

Homology relating class I aaRS to class II aaRS is shown in Figure 8. A schematic alignment is shown. Because class IA and class IIA aaRS have alternate folds, for the most part, active site β-sheets do not align. At the base of aaRS evolution, a shared Zn finger is identified. The extended N-terminus of class I aaRS and the N-terminal Zn finger direct the alternate folding of class I aaRS and prevent a class II aaRS fold [2,5].

Because of alternate folds, Phyre2 cannot be used to obtain a class I and class II aaRS alignment. Previously, the best (lowest) e-value we could obtain for apparent homology of class I and class II aaRS enzymes was 0.001 (i.e., about a 1:1000 chance of apparent homology being due to random chance) [2]. Therefore, we collected sets of GlyRS-IIA, IleRS-IA, and ValRS-IA enzymes from ancient archaeal species. We then did the pairwise alignments expecting to find lower e-value scores. Ranking the output by lowest e-value, the best scores obtained were for *Methanobacterium congolense* GlyRS-IIA and *Methanobacterium bryantii* IleRS-IA (e-value 4.6 × 10^−11^) (the highest apparent homology detected for GlyRS-IIA to IleRS-IA) and *Candidatus Methanoperedens nitroreducens* GlyRS-IIA and *Methanococcoides burtonii* ValRS-IA (e-value 1.3 × 10^−8^) (the highest apparent homology detected for GlyRS-IIA to ValRS-IA). We conclude that GlyRS-IIA and IleRS-IA and GlyRS-IIA and ValRS-IA are homologs, and that class IA enzymes probably derived from the in-frame N-terminal extension and refolding of an early evolutionary version of GlyRS-IIA. We posit that class IIA aaRS enzymes derive from GlyRS-IIA, and class IA aaRS enzymes derive from GlyRS-IIA, apparently rooting the lineages of class I and class II aaRS enzymes. It appears to us that all aaRS enzymes, therefore, derive from a primordial version of GlyRS-IIA, consistent with glycine being the primordial amino acid in the code.

Contrary to an alternate model [52,53,54], class I and class II aaRS enzymes do not derive from an ancestral bidirectional gene [2]. Rather, class I and class II aaRS enzymes are homologs (Figure 8). In this report, we trace the divergence of class I and class II aaRS enzymes using the Phyre2 online server (Figure 6 and Figure 7). We find that the evolutionary paths for aaRS divergence can be determined with little ambiguity. In order to build accurate alignments among structurally-related proteins, Phyre2 is remarkably sensitive when compared to standard sequence alignments. Many comparisons that were tenuous based on sequence alignments, could be unambiguously determined or falsified using Phyre2 (Figure 6 and Figure 7). A slight drawback is that Phyre2 is limited to searching the Protein Data Bank and may overlook some closer sequence homologies that are not represented in a limited and biased set. To identify more optimal sequence matches, therefore, searches must be done using other methods (i.e., Figure 8). A convenient method to find closest homologs of distantly related proteins using the sequence data bases would be a useful complement to Phyre2.

### 6.4. Coevolution of aaRS Clades and the Genetic Code

**Figure 9:**
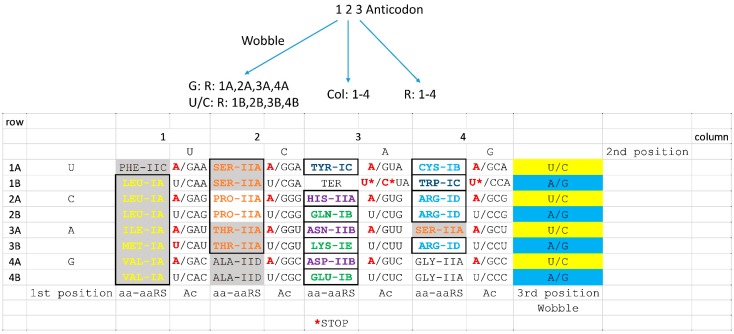
Closely related aaRS enzymes align in columns of the genetic codon-anticodon table demonstrating coevolution of aaRS enzymes and the genetic code. The diagram above the figure indicates how genetic code columns and rows relate to the tRNA anticodon. The table is drawn to emphasize the code in archaeal systems, which are close to the base of code evolution. The code is effectively a 32-letter code in archaea, limited by tRNA anticodon wobble ambiguity. Bases in red type are not utilized in archaeal tRNAs [2]. Grey shading indicates aaRS enzymes that edit inappropriately attached amino acids. aaRS enzymes that are related are indicated by common color-shading and/or boxing. Closest aaRS homologs are often observed within columns. Ac, anticodon; aa-aaRS, amino acid-aaRS subclass; R, row; Col, column. See the text for details. The figure is adapted and improved from reference [5] with new aaRS homology data.

At the base of genetic code evolution, the code should be viewed as a 32 letter code, because the code is limited to 32 letters in tRNA (Figure 9). By contrast, all codons are represented in mRNA. As noted above, the code is limited in tRNA because only pyrimidine versus purine resolution is achieved at the anticodon wobble position. We posit that the code evolved and structured around the tRNA anticodon according to rules for anticodon interpretation on the ribosome. Because of the importance of the tRNA anticodon, the code is shown as a codon-anticodon table. Bases that are not utilized in archaea are indicated in red, demonstrating that the code is reduced in tRNA relative to mRNA. In this review, we present and support a tRNA- and anticodon-centric view of genetic code evolution.

We have previously indicated apparent coevolution of tRNAs, amino acids, the genetic code, and aaRS enzymes. One of the primary models for evolution of the code is the coevolution theory that posits that amino acids, the genetic code, tRNAomes, ribosomes, and aaRS enzymes are coevolved [27,28,55,56]. Here, by better determining the homologies of aaRS enzymes, we support the coevolution theory in additional detail. We have previously determined that the genetic code appears coevolved primarily within columns (Figure 9) [2,5]. Because columns represent position 2 of the tRNA anticodon, and because position 2 is the most important position for translational accuracy [44,46,47], the primacy of tRNA anticodon position 2 appears to explain coevolution of the code within columns. Here, we extend the idea of the primacy of the second anticodon position. In column 1 of the code, ValRS-IA, IleRS-IA, LeuRS-IA, and MetRS-IA are closely related enzymes, and Val, Ile, Leu, and Met are closely related hydrophobic amino acids. In column 2, ProRS-IIA, ThrRS-IIA, and SerRS-IIA are closely related enzymes, and Pro, Thr, and Ser are related and neutral amino acids (properly, Pro is referred to as an imino acid). Thr and Ser are closely related amino acids. In column 3, AspRS-IIB, AsnRS-IIB, and HisRS-IIA are related enzymes, and Asp and Asn are closely related amino acids. AspRS-IIB and AsnRS-IIB are very closely related enzymes. Also in column 3, GluRS-IB, LysRS-IE, and GlnRS-IB are related enzymes, and Glu and Gln are closely related amino acids. Also, Asp, Asn, and His utilize tRNA anticodon wobble G (column 3, rows 4A, 3A, and 2A) (in archaea anticodon wobble A is not utilized [2,35]), and Glu, Lys, and Gln utilize anticodon wobble U/C (column 3, rows 4B, 3B, and 2B), further supporting coevolution of aaRS enzymes and tRNAs within column 3. *Pyrococcus furiosis* (used here as the reference species for most queries using Phyre2) and many other ancient archaea lack GlnRS-IB. Instead, these organisms charge tRNA^Gln^ with Glu and recruit an enzyme (GatE) to catalyze amination of Glu to Gln. In column 4, ArgRS-ID and CysRS-IB are related enzymes. TyrRS-IC and TrpRS-IC appear to possibly derive from an ancestor of CysRS-IB, indicating late evolution of the genetic code across row 1 rather than within columns. By the time of Tyr and Trp addition, code columns and favored rows were mostly occupied with other amino acids. We conclude that the genetic code remains highly structured according to its original coevolution pathway, and, to a startling extent, the pathway of code coevolution remains apparent and centered on the tRNA anticodon and anticodon sequence preferences.

## 7. Coevolution of tRNAomes and the Genetic Code

**Figure 10:**
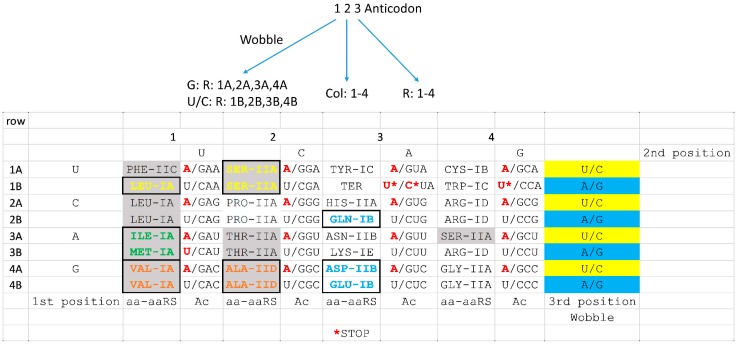
Early tRNAomes evolved within columns and within rows across neighboring columns. The diagram above the figure indicates how genetic code columns and rows relate to the tRNA anticodon. In *Pyrococcus* species, tRNA^Leu^ and tRNA^Ser^ are closely related. tRNA^Ile^ and tRNA^Met^ (one initiator and two elongator tRNA^Met^) are closely related. tRNA^Val^ and tRNA^Ala^ are closely related and tRNA^Asp^, tRNA^Glu^, and tRNA^Gln^ are closely related. Colors and boxing are used to emphasize related tRNAs within columns or within rows across neighboring columns. Grey shading indicates aaRS with editing active sites.

tRNAomes in *Pyrococcus* species are similar to a LUCA tRNAome, but, with subsequent evolution, tRNAomes become more chaotic and more difficult to trace to their roots [1]. In Figure 10, sectoring of the genetic code is analyzed in terms of tRNAomes in ancient archaea. Notably, closely related tRNAs are found within columns, consistent with aaRS evolution (Figure 9), and, also, within rows across neighboring columns. Within column 1, tRNA^Ile^ and tRNA^Met^ are closely related tRNAs in *Pyrococcus*. In *Pyrococcus*, there are two elongator tRNA^Met^ and one initiator tRNA^Met^. These closely related tRNAs are distinguished by distinct tRNA modifications [57,58]. Within column 3, tRNA^Asp^, tRNA^Glu^, and tRNA^Gln^ are closely related tRNAs, supporting coevolution of tRNAs and amino acids within column 3. Because tRNA anticodon position 2 is most important for translational accuracy, tRNA evolution is also expected across neighboring columns but within rows. Discrimination of closely related tRNAs is easiest with a change in the base at anticodon position 2. In keeping with this expectation, type II tRNAs, tRNA^Leu^ and tRNA^Ser^ (row 1; columns 1 and 2), are closely related, and tRNA^Val^ and tRNA^Ala^ (row 4; columns 1 and 2) are closely related in *Pyrococcus*. We consider close relatedness of tRNAs in neighboring sectors of the genetic code to support coevolution of tRNAs, amino acids, aaRS enzymes, and the genetic code.

Furthermore, the code structure is best represented using the table format shown in Figure 9 and Figure 10 rather than an alternate display (i.e., a circular code diagram [59]). We previously showed that aaRS editing of inappropriately joined amino acids, by a separate editing active site, is mostly limited to the left half of the archaeal genetic code table [2,4,5]. SerRS-IIA is located in both the left and right halves, but Ser is the only amino acid split between the left and right halves of the table. Furthermore, Ser is the only amino acid found within fully disconnected sectors of the code. We note that Ser (anticodon GCU) may have jumped to occupy a favored sector of the code (column 4; second anticodon position C). It appears that the first four amino acids in the code (i.e., Val, Ala, Asp, and Gly) may have ended up residing in row 4 of the code, indicating further structure. Preference for row 4 of the code appears to reflect a selection for third position C in the tRNA anticodon. The third anticodon position is the second most important position for translational accuracy [44,46,47]. Generally, C appears to be favored in the second and third anticodon positions, indicating that a tRNA anticodon with a small base (C or U) that makes three hydrogen bonds (C not U) is favored. We posit that anticodon U (pyrimidine, two hydrogen bonds) and G (purine, three hydrogen bonds) are similarly favored in evolution of the code. With current knowledge, we cannot judge whether there is a slight anticodon preference for U or G. By contrast, A appears to be disfavored in the anticodon because A is a purine that forms only two hydrogen bonds. Interestingly, in archaea and bacteria, A is also strongly disfavored in the anticodon wobble position [2,35], consistent with bulky A being negatively selected in the anticodon loop. Favoring of anticodon C in the second and third positions could explain why Gly, which is posited to be the first encoded amino acid, occupies row 4 and column 4 of the table (Figure 9 and Figure 10).

Based on similar reasoning, third anticodon position A (row 1) is expected to be a less favorable position of the genetic code table. Consistent with this assessment, Phe, Tyr, Trp, and Cys, which are some of the last encoded amino acids, are located in row 1. These amino acids, along with His and Met, are thought to be among the final additions to the genetic code table [1,2]. Stop codons, which are read by proteins, not tRNAs, and are not bound by tRNA preference rules, are found in disfavored row 1.

At the base of genetic code evolution, the code in archaea is effectively a 32-letter code (in tRNA) as opposed to a 64-letter code (in mRNA) [2,4,5]. The code is limited to a maximum of 32 letters in tRNA because, reading mRNA codons, tRNA cannot adequately discriminate single bases (A, G, C, and U) at the anticodon wobble position. Rather, tRNAomes initially evolved with effective purine/pyrimidine discrimination at the anticodon wobble position, which is the first position in the anticodon (Figure 5). Therefore, mRNA wobble A and G are read ambiguously by tRNA wobble C/U, and mRNA wobble U and C are read ambiguously by tRNA wobble G. Anticodon wobble A is rarely used in archaea [2,35]. Because the code is effectively smaller in tRNA than in mRNA, the genetic code in Figure 9 and Figure 10 is represented as a 32-letter code and as a codon-anticodon table, to stress the central importance of the anticodon. We find other representations of the genetic code table to be overly complicated and misleading. The 64-letter representations of the code fail to recognize tRNA anticodon wobble ambiguity, so showing the 32-letter code is simpler, yet complete. Because the code is limited in tRNA, not mRNA, showing the code in terms of anticodons is essential. Other representations obscure simplicity of the code, structure in the code and pathways of code coevolution.

## 8. Evolution of the Genetic Code

**Figure 11:**
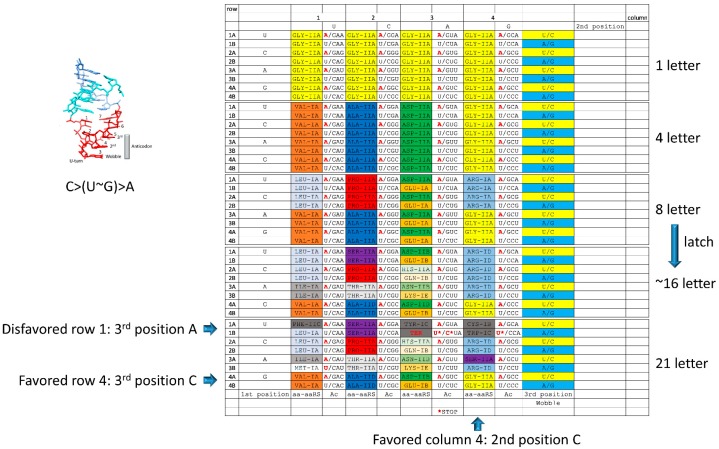
A detailed working model for evolution of the genetic code. Shading colors help to indicate modes of occupancy for amino acids.

Because the genetic code evolved around tRNAs, the pathway for evolution of the code was organized by the tRNA anticodon (Figure 5). The central position of the anticodon (position 2) is the most important for translational accuracy [44,46,47]. In the genetic code table (i.e., Figure 9 and Figure 10), anticodon position 2 is represented by genetic code columns. From a one-letter code in which all anticodons encode Gly, the code is posited to have evolved to encode Val, Ala, Asp, and Gly, along genetic code columns 1–4 (Figure 11) [2,27].

Next, the code is posited to have sectored primarily on the third anticodon position, which is second most important for translational accuracy. Initially, purine versus pyrimidine resolution was achieved at the third position for columns 1, 2, and 4, encoding Val, Leu, Ala, Pro, Gly, and Arg. By contrast, column 3 is posited to have divided along the anticodon wobble position to encode Asp in column 3, rows 4A, 3A, 2A, and 1A and Glu in column 3, rows 4B, 3B, 2B, and 1B.

In order to achieve single base recognition at the third anticodon position, and in order to read the wobble position, enhanced accuracy was required in ribosome recognition of the tRNA anticodon. The tRNA-aa enters the ribosome in complex with G-protein GTPase EF-Tu. The ribosome and EF-Tu·GTP→GDP·tRNA-aa·mRNA forms a “latch”, tightening the codon-anticodon interaction and enhancing translational accuracy. In the *Thermus thermophilus* ribosome, the latch is formed of invariant residues 16S rRNA G530, A1492 and A1493 and 23S rRNA A1913 [44,46,47]. The closed latch enforces Watson–Crick geometry on the second and third anticodon positions, allowing single base accuracy in reading positions 2 and 3. By contrast, at the wobble position, single base accuracy is achieved only with difficulty and essentially never at the base of code evolution. Evolution of the latch on the ribosome may have allowed further evolution of the code and a transition from an ~8-letter code to an ~16-letter code (Figure 11).

Once single base resolution was achieved at the third anticodon position via evolution of the EF-Tu codon-anticodon latch, columns sectored further. In column 1, Ile was added, replacing Val in rows 3A-B. In archaea and bacteria, Ile (anticodon GAU) and Met (CAU) are used almost exclusively [2]. In column 2, Thr (rows 3A-B) and Ser (rows 1A-B) were added. In column 3, Asn replaced Asp in column 3, row 3A. His replaced Asp in column 3, row 2A. Lys replaced Glu in column 3, row 3B. Gln replaced Glu in column 3, row 2B.

To complete the code sectoring, Met (CAU) occupied column 1, row 4B. Phe (GAA) occupied column 1, row 1A. In column 3, Tyr (GUA) occupied column 3, row 1A. In column 4, Arg occupied column 4, row 3B and Ser (GCU) occupied column 4, row 3A. Ser may have invaded column 4, row 3A, to access a more favored sector in the code (second position C) (see below). Ser is the only amino acid in the code with disconnected sectors (split between columns 2 and 4). Cys occupies column 4, row 1A. Trp occupied column 4, row 1B.

According to the model (Figure 11), when sectors were occupied, another amino acid was displaced from the position, and an apparent hierarchy of favored positions in the tRNA anticodon is observed. Notably, in the second and third anticodon positions, the tRNA anticodon appears to favor pyrimidines over purines, and bases forming three hydrogen bonds (C and G) are favored over those that form two hydrogen bonds (U and A). The observed pattern, therefore, is C>(U~G)>A. Amino acids that previously occupied the code appear invariably to retain favored positions and to yield less favored anticodon positions, giving insight into the probable order of amino acid additions to the code. According to this ranking of anticodons, the most favored position in the code is column 4 (second position C) and row 4 (third position C), which is held by Gly, posited to be the primordial encoded amino acid. Because row 4 (third position C) is favored, Val, Ala, Asp, and Gly (thought to be the first 4 amino acids in the code) defended these favored sectors. The last amino acids added to the code, i.e., Phe, Tyr, Trp, and Cys, are found in disfavored row 1 (third position A). Stop codons, which are recognized by proteins and not tRNAs and, therefore, are independent of tRNA anticodon preference rules, occupy disfavored row 1 (third position A).

Thus, a highly detailed working model can be constructed for evolution and sectoring of the genetic code based on a hierarchy of favored tRNA anticodon positions (C>(U~G)>A). By this reasoning, positions of every amino acid in the code can be rationalized according to rules for Darwinian selection. A is most strongly disfavored, resulting in the final amino acid additions and stop codons in row 1 (third position A). Interestingly, A is also strongly disfavored in the anticodon wobble position in archaea and bacteria [2,35]. In bacteria, A is only encoded when it is modified by deamination to inosine (i.e., Arg (ACG→ICG) (I for inosine)), consistent with A being disfavored in other anticodon positions. Of course, A is required in anticodon positions 2 and 3 in order to fill in the genetic code table and to evolve a complex code.

Intriguingly, hydrophobic amino acids tend to locate to column 1 and neutral amino acids to column 2. Evolution of the genetic code within columns reflects the primacy of the second anticodon position. Hydrophobic and neutral amino acids, furthermore, tend to be found in four-codon sectors. aaRS editing of incorrectly attached amino acids on tRNA is also a feature of columns 1 and 2 of the code. It appears that hydrophobic and neutral amino acids with no charge and little capacity for hydrogen bonding cause some difficulties for accurate attachments to their cognate tRNAs, and may, therefore, require a four-codon sector to reduce ambiguity in tRNA charging and may also require aaRS editing to reduce tRNA charging errors. Maintenance of four-codon sectors limits the coding capacity of the genetic code, but it appears that some four-codon sectors cannot easily be split into two two-codon sectors without an insupportable error catastrophe in translation, perhaps because of the nondescript identities of the amino acids that are encoded [2].

Thus, the genetic code broke into sectors around the tRNA anticodon. Sectoring around the anticodon is evident for the second position (columns 1–4), third position (rows 1 and 4) and wobble position (column 3). The characteristics of amino acids affected the sectoring of the code. Because of coevolution, hydrophobic amino acids are located in column 1. Neutral amino acids are located in column 2. Many aaRS enzymes associated with columns 1 and 2 edit inappropriately attached amino acids. Column 3 is most highly sectored and encodes amino acids with charge or multiple hydrogen bonds, facilitating the accuracy of tRNA charging by aaRS enzymes and facilitating code sectoring into two-codon sectors. Column 3 also appears to have been sectored by a different mechanism than columns 1, 2, and 4. In column 4, Arg has significant character, i.e., Arg is bulky with diffused charge and multiple hydrogen bonding groups, and Gly is the smallest amino acid. Consistent with more distinct amino acid character, aaRS enzymes from columns 3 and 4 do not edit in archaea because, for the most part, accurate attachment of amino acids in columns 3 and 4 is not as challenging as it is for amino acids encoded in columns 1 and 2. We posit that these challenges to distinguish hydrophobic and neutral amino acids in columns 1 and 2 are met by: (1) aaRS editing; and (2) maintenance of four-codon sectors. aaRS editing, amino acid character and identity, and maintenance of four-codon sectors are issues of translational fidelity, so patterns of sectoring of the genetic code are a compromise between code complexity, amino acid identities, and minimization of translation errors [2].

As noted above, column 3 is posited to have sectored via a slightly different mechanism than columns 1, 2, and 4, because column 3 appears to have sectored around the anticodon wobble position rather than position 3. Here, we favor modeling the initial sectoring, by allotting Asp to column 3, rows 4A, 3A, 2A, and 1A (around wobble position G) and Glu to column 3, rows 4B, 3B, 2B, and 1B (around wobble position C/U). If the proposed sectoring mechanism is correct, apparent sectoring of column 3 around the anticodon wobble position is explained.

## 9. The tRNA-Centric View

Remarkably, a clear record is maintained in archaea of the tRNA^Pri^ sequence, which was formed of repeats and inverted repeats (Figure 1, Figure 2, Figure 3 and Figure 4) [1,2,3,4]. Because tRNA^Pri^ was generated via abiogenesis, evidence emerges from inspection of tRNA sequences for a polymer world and minihelix world with unexpected order, preceding cellular life. Thus, analysis of existing sequences provides surprising insights into abiogenic processes from ~4 billion years ago.

A clear record of the evolution of type I and type II tRNAs by processing of a common 93-nucleotide precursor is documented (Figure 1, Figure 2, Figure 3 and Figure 4). Type I and type II tRNAs are the crowning achievements of abiogenesis, because, once tRNAs evolved, a Darwinian selection path was available for evolution of mRNA, aaRS enzymes, translation systems, and the genetic code, leading to cellular life. Coevolution of aaRS enzymes, tRNAs and the genetic code is apparent. Evolution of homologous aaRS enzymes and related amino acids within genetic code columns demonstrates the primacy of the second position of the tRNA anticodon because genetic code columns represent the second position of the anticodon (Figure 9 and Figure 10). Position 2 of the anticodon is also the most important for translational accuracy. Evolution of the genetic code can be rationalized according to a C>(U~G)>A sequence preference for the tRNA anticodon positions 2 and 3. Why tRNAs appear to demonstrate these preferences is not clear. Preference for C and G can be rationalized because these bases form three hydrogen bonds, making the codon-anticodon interaction stronger. Selection for C and U may reflect a positive selection for small base size in the anticodon. If small bases are favored in the anticodon, molecular dynamics simulations of latched anticodon loop structures and mRNA codons may provide insight. Of course, an apparent preference for small bases in the tRNA anticodon may rather represent a preference for large bases in mRNA codons. Also, such a sequence preference in tRNA or mRNA may no longer apply because of evolution of features such as the ribosome EF-Tu latch.

Location of Gly to column 4, row 4 (second and third position C), is consistent with Gly being the primordial encoded amino acid, as indicated in Figure 1, Figure 2, and Figure 4, and, as has also been posited by others [1,2,4,5,48,49,50]. Location of Val, Ala, Asp, and Gly, which are likely the first four encoded amino acids [27], to row 4 (favored third anticodon position C), is also consistent with a tRNA-centric view of coevolution. Phe, Tyr, Cys, and Trp are four of the last amino acids encoded, and these amino acids are relegated to the least favored row 1 (third anticodon position A) (Figure 11). Stop codons, which are recognized by proteins, not tRNAs, and are, therefore, not bounded by anticodon preferences, are also relegated to disfavored row 1, as expected. The genetic code, therefore, evolved around the tRNA anticodon by a predictable pathway. aaRS enzymes coevolved with tRNAomes and the genetic code (Figure 9 and Figure 10). The patterns of code evolution are apparent in the patterns of aaRS coevolution and the coevolution of related amino acids. Interpreting tRNAome evolution is somewhat more challenging, but, in ancient archaea, before extensive radiation and often convergence of tRNA sequences, a few examples of tRNA relatedness within columns and a few examples of tRNA relatedness within a row and across neighboring columns are evident.

## 10. Simulation of Genetic Code Evolution

Computer simulations of genetic code evolution have mostly been done using a 64-codon mRNA code. As we have explained, at the base of code evolution, the genetic code should be considered to be a 32 letter code because of tRNA anticodon wobble ambiguity reading mRNA on the ribosome [2,5]. Thus, tRNA, not mRNA, limits the capacity for coding. Viewing the initial code as, at most, 32 letters shrinks the problem of genetic code evolution and renders simulating evolution of the code a more manageable computational problem. Here we describe many of the selective pressures for building up and structuring the genetic code. Very strong correlations are shown that shape coevolution of aaRS enzymes and the tRNA anticodon. Furthermore, some weaker rules appear to shape the divergence of tRNAs and the initial establishment of tRNAomes. In aaRS evolution, there is clear evidence for coevolution of aaRS enzymes, amino acids, tRNAs, and the genetic code. Centering the view on tRNA and the tRNA anticodon significantly simplifies understanding of genetic code evolution.

## 11. Evolution of the Genetic Code as an Artificial Intelligence Problem

Evolution of the genetic code is a fundamental artificial intelligence problem and should be thought of and approached as such. Simply stated, through evolution, the genetic code teaches itself (“learns”) to encode proteins. The ability to encode proteins enables the complexity of modern biology. The genetic code adds amino acids via tRNA charging errors [2]. As translational fidelity mechanisms evolve, the structure of the code becomes ever more stable. The genetic code evolves toward maturity and closure because of the development of translational fidelity mechanisms, so there is a limit to evolved code complexity (i.e., 20 amino acids + stops). This limit is indicated by establishment and maintenance of four-codon sectors, which limit the complexity of the final code [2]. Some four-codon sectors may be difficult to split because the amino acids encoded (i.e., hydrophobic and neutral with little capacity for forming hydrogen or ionic bonds) are more difficult to discriminate within an aaRS active site. Once the standard genetic code is established with many coevolved and reliant systems, many further modifications became lethal. Because the code appears to have evolved rapidly as a “frozen accident”, much of the initial code structure is maintained, and a record of code evolution is largely conserved.

## 12. Life on Earth

**Figure 12:**
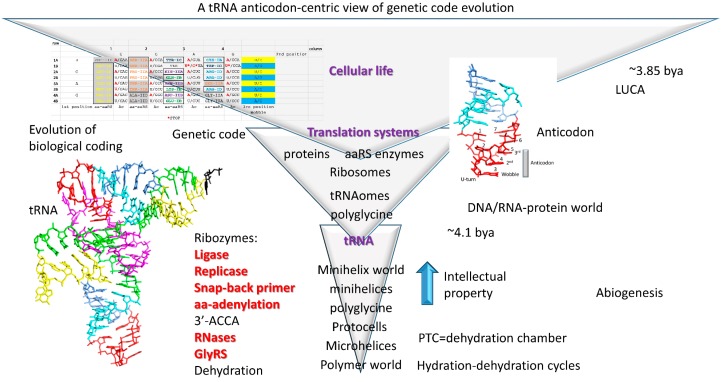
Evolution of life on Earth is centered on abiogenesis of tRNA. Repeat and inverted repeat sequences, identified in archaeal tRNAs, and a small number of ribozymes appear sufficient to generate tRNA, which appears sufficient to generate the genetic code and translation systems leading to cellular life. Ribozymes indicated in red have been generated in vitro. PTC, peptidyl transferase center. Ancient polymerization reactions may have been driven by hydration–dehydration cycles [5]. Triangles indicate increases in biological complexity with enhanced evolutionary innovation.

Life on Earth evolved around tRNA, making tRNA evolution the most important and central event in evolution of living systems (Figure 12). A clear path to tRNA evolution is described from repeating sequences and stem-loop-stems that, remarkably, are conserved in tRNAs of ancient archaea (Figure 1, Figure 2, Figure 3 and Figure 4). tRNA^Pri^ is the product of abiogenesis. A small number of ribozymes, essentially all of which have been generated in vitro [11,12,13,16,17,18,42,60], appear sufficient to generate tRNA. Once tRNA^Pri^ evolves, tRNAomes, mRNAs, a genetic code, aaRS enzymes, ribosomes, and translation systems became inevitable. The tRNA anticodon is a central feature of genetic code evolution. Genetic code tables must be rendered as anticodon tables because of the central importance of tRNA and the tRNA anticodon. The complexity of the genetic code was determined by tRNA not mRNA. The genetic code, tRNAomes, aaRS enzymes, amino acids, mRNA, ribosomes, and translation systems coevolved, but tRNA was the central innovation and driver of that coevolution. After these innovations, the LUCA and cellular life became inevitable [5]. tRNA, therefore, is the founding molecule for evolution of living systems on Earth. To generate a living system on another planet would require evolution of tRNA or an analogous molecule around which to nucleate a genetic code. Without the innovation of tRNA, no complex biology appears possible.

## Figures and Tables

**Table 1:** Phyre2 homology scores for class II aaRS. ND, not detected; NR, not reported. Not reported scores (i.e., GlyRS-IIA versus GlyRS-IIA) are similar to the highest scores in the table.

	SerRS-IIA	ThrRS-IIA	ProRS-IIA	GlyRS-IIA	HisRS-IIA	AspRS-IIB	AsnRS-IIB	LysRS-IIB	PheRS-IIC	pSerRS-IIC	AlaRS-IID	pyrroLysRS-IIC	GlyRS-IID	target
SerRS-IIA	NR	412	384	275	159	58	59	74	77	81	26	156	ND	
ThrRS-IIA	412	NR	540	554	456	80	ND	75	77	69	ND	129	ND	
ProRS-IIA	373	540	NR	495	378	63	ND	70	96	79	ND	127	ND	
GlyRS-IIA	307	554	608	NR	392	63	ND	98	79	71	ND	141	ND	
HisRS-IIA	149	456	339	393	NR	79	ND	181	104	83	17	123	ND	
AspRS-IIB	68	80	71	57	156	NR	807	816	179	98	37	224	36	
AsnRS-IIB	59	ND	ND	ND	ND	807	NR	778	98	81	33	216	ND	
LysRS-IIB	69	75	75	66	180	815	778	NR	180	101	30	265	33	
PheRS-IIC	72	77	69	60	64	107	98	106	NR	719	37	203	37	
pSerRS-IIC	75	69	75	61	72	99	81	102	721	NR	32	321	32	
AlaRS-IID	ND	ND	ND	ND	ND	36	33	ND	37	32	NR	37	597	
pyrroLysRS-IIC	103	129	124	107	118	229	216	243	350	335	37	NR	37	
GlyRS-IID	ND	ND	ND	ND	ND	36	ND	33	40	36	596	37	NR	
query														

**Table 2:** Phyre2 homology scores for class I aaRS. NR, not reported. Not reported scores are similar to the highest scores in the table. All class I aaRS enzymes are connected by detectable homology using Phyre2.

	IleRS-IA	LeuRS-IA	MetRS-IA	ValRS-IA	ArgRS-ID	TrpRS-IC	TyrRS-IC	CysRS-IB	GluRS-IB	GlnRS-IB	LysRS-IE	target
IleRS-IA	NR	1449	846	1508	259	50	55	301	113	137	73	
LeuRS-IA	1347	NR	780	1331	250	38	47	332	115	129	73	
MetRS-IA	817	774	NR	805	349	52	35	435	156	126	159	
ValRS-IA	1663	1322	854	NR	259	50	55	301	118	154	73	
ArgRS-ID	261	274	370	266	NR	56	58	348	151	100	196	
TrpRS-IC	34	38	68	34	52	NR	495	54	69	44	36	
TyrRS-IC	33	29	33	25	41	485	NR	57	54	40	42	
CysRS-IB	353	394	446	360	387	64	83	NR	258	293	220	
GluRS-IB	59	86	139	61	121	65	67	167	NR	1203	331	
GlnRS-IB	59	72	126	63	123	47	48	156	1538	NR	313	
LysRS-IE	181	209	234	189	249	67	73	330	549	281	NR	
query												

## Abbreviations

aaRS	Aminoacyl-tRNA synthetase (i.e., GlyRS)
Ac loop	Anticodon loop
LUCA	Last universal common (cellular) ancestor
NCBI	National Center for Bioinformatics Information
PTC	Peptidyl transferase center
T loop	T loop or TΨC loop
V loop	Variable loop
